# On dueling multi-act arithmetic: exploring the dynamics of goal-driven competition on engagement and cognition

**DOI:** 10.1007/s00221-025-07175-9

**Published:** 2025-10-21

**Authors:** Michael B. Steinborn, Lynn Huestegge

**Affiliations:** Department of Psychology III, Julius-Maximilians-Universität Würzburg, Röntgenring 11, Room H7, 97070 Würzburg, Germany

**Keywords:** Introspective self-report, Mental arithmetic, Social facilitation, Effort mobilisation, Meta cognition, Speed-accuracy trade-off

## Abstract

Ludic design in mental chronometry seeks to enhance engagement through socio-interactive elements. The present study examined whether a co-actor duel context influences both performance and subjective experience. Participants completed speeded arithmetic at two difficulty levels (easy, hard) under two context conditions (alone, duel) in a mixed within-subject design. Self-reports of engagement, distress, and worry were obtained before and after tasks. In the duel context, participants completed problems more quickly, accompanied by a small rise in errors, which however, remained far below the 10% margin allowed by duel rules, indicating that the increase was not a deliberate sacrifice of accuracy for speed. We interpret the speed-up as improved efficiency with preserved engagement, where the modest error rise reflects the probabilistic cost of reduced checking time rather than relaxed accuracy criteria. Such minor differences are unlikely to be consciously detected and therefore are not introspectable as a performance decline.

**Supplementary Information:**

The online version contains supplementary material available at 10.1007/s00221-025-07175-9.

## Introduction

Ludic design in mental chronometry integrates socio-interactive elements into contexts that lack inherent playfulness or motivation, with the aim of enhancing engagement and drive (Strayer et al. 2024; Strobach and Huestegge 2017; Welhaf 2024; Wolf et al. 2025). Common techniques include: *(a)* reward systems linked to social comparison, such as points, badges, or leaderboards, to stimulate strategic motivation; *(b)* goal-setting features, such as challenges or quests, to strengthen volition and tactical engagement; and *(c)* ludic perceptual feedback that provides a pleasant sensory experience, fostering a sense of agency (Barsalou 1999; Dourish 2001; Greenwald 1970; Haggard 2008; Verbeek 2005). Interest in socio-interactive engagement dates back to the early twentieth century (Allport 1920; Burnham 1910; Dashiell 1930). Notably, Thorndike (1922) examined social-context effects in mental arithmetic, contrasting individual with group work and competitive with cooperative settings. He argued that competition may impede skill acquisition (e.g., learning arithmetic facts or developing novel strategies) yet enhance the application of established skills (e.g., strengthening arithmetic fluency). Building on this tradition, the present study examines whether gamified competition in speeded mental arithmetic, implemented as a simulated cognitive arena or “brain-brawl zone”, can enhance performance and subjective engagement. By analysing how socio-interactive features shape goal representation and execution in a cognitive task, we seek to elucidate how competition modulates both experienced engagement and observable outcomes.

### Social-context effects on performance: benefits and costs

The effect of a social context, such as a mental-arithmetic competition, is typically examined by contrasting a control condition (alone or single-context) with a critical condition (presence or social-context). In the literature, this set-up appears under umbrella terms such as the co-actor paradigm, the audience paradigm, or the mere-presence paradigm (Böckler et al. 2012; Götz et al. 2023; Krishna and Götz 2024; Steinborn and Huestegge 2017, 2020; Wühr and Huestegge 2010). Specific implementations vary depending on whether the aim is to study socio-motor interaction or evaluation (Götz and Dreisbach 2024; Kunde et al. 2018; Wiese et al. 2017), or to isolate the alerting response elicited by the simple presence of an attendant confederate (Steinborn and Huestegge 2017, 2020). Historically, social-context effects have a long tradition in experimental psychology. Triplett (1898) famously reported that cyclists rode faster when racing together than when racing alone. Such contexts involve multiplex mechanisms, with both facilitation and inhibition observed (Bond and Titus 1983; Guerin 2009). Another historical turning point is the study of Allport (1920) who examined whether the presence of a group of other individuals (working on the same task) affects the swiftness and exactness of generated verbal associations. Actually, the individuals formed a greater number of associations when in the presence of the confederates as compared to when they were alone (Burnham 1910; Church 1962; Dashiell 1930).

Many sports and games are primarily recreational, even when inherently competitive. Competitive activities are often pursued in leisure time because they create distance from everyday routines and restore attention by shifting from satiated routines to exploratory modes. This change enhances engagement through gamified mini-goals and feedback loops that maintain motivational commitment, tactical focus, and ludic inspiration. The literature documents that competition can facilitate or inhibit performance depending on the task (Bond and Titus 1983; Geen and Gange 1977). Zajonc (1965) proposed that mere presence facilitates simple tasks but impairs complex ones, even without direct interaction or authority (Brewer and Ridgway 1998; Götz et al. 2023; Steinborn and Huestegge 2017, 2020). His definition of complexity, however, was taxonomic, not operational, overlooking differences in the mental operations required. For example, simple tasks were often defined as speed tests (e.g., hammering nails for one minute) and difficult tasks as power tests (e.g., using a hammer to solve a novel problem) (e.g., Bartis et al. 1988; McGraw 1978; Zajonc 1965). More recent studies employ chronometric tasks, where item difficulty can be precisely varied, especially in arithmetic, and where highly reliable performance measures are available (Klauer et al. 2008; Sharma et al. 2010; Steinborn and Huestegge 2020).

An alternative account, proposed by Sanders and Baron (1975), holds that the presence of a social agent, whether spectator or co-actor, elevates a general level of non-specific readiness. The presence of others is assumed to trigger a socio-motor tendency to interact, that is to receive and transmit information, which may be beneficial or distracting depending on the information’s relevance (Kunde et al. 2018; Liepelt 2014; Vesper et al. 2017). Co-working individuals can increase global alertness but also heighten susceptibility to distraction, whether from external events or from their consequences. Scheier and Carver (1977) argued that social presence intensifies self-focus through social comparison. In such situations, individuals monitor cues about their relative standing, which can disrupt task processing. Increased arousal from these comparisons can also distort mental representations of the distribution of monitored events, potentially undermining cognitive efficiency (Brewer 1995; Brewer and Ridgway 1998; Deutsch et al. 2023; Hasher and Zacks 1979; Mussweiler and Strack 2000). Social presence thus creates two principal distraction sources: monitoring the external environment and processing internal evaluations (Wells and Matthews 2015, chap. 12).

### Monitoring versus focusing: spare–utilized capacity threading

Integrating theoretical ideas with empirical findings suggests that social facilitation reflects a moderate alertness response induced by the presence of others, subjectively experienced as increased readiness (Gerald Matthews 2021; Steghaus and Poth 2024; Thayer et al. 1994). This socially alerted cognition supports the sustained mobilisation of effort (Dietze et al. 2023; Steinborn et al. 2017; Strayer et al. 2024). Social inhibition, in contrast, can be understood as transient performance decline arising from two principal sources: event distraction and self-referential processing (Wells and Matthews 2015, chap. 12). Such distractions occur intermittently, during moments of attentional capture or evaluative processing (Steinborn et al. 2017; Unsworth and Robison 2020), and reflect temporary lapses rather than a global slowing of mental operations. The resulting performance decline is assumed to originate not from a global slowing of mental operations but from an increased likelihood of attentional lapses during the task; thus it is crucial to consider measures of performance fluctuations (Sharma et al. 2010; Steinborn and Huestegge 2020).

A *spare–utilized capacity threading model* makes the structural assumption that resources are limited in two categorical ways, according to Schumann et al. (2022a, b) how focal attention is allocated at one defined moment in time, and in how resources can be distributed across a series of consecutive moments (e.g., a time span or work period). Regardless of task complexity, individuals can engage fully in one focused activity only for a brief period (Kahneman and Beatty 1966; Van Breukelen et al. 1995), after which renewed acts of intention are required to sustain high-level processing (Steinborn et al. 2017). Attention is therefore not maintained by an “*autopilot*” homunculus, but must actively re-implemented by wilful acts of re-transforming spare to utilized capacity. Critical is the distinction between the rate of utilized versus spare capacity as individuals are assumed to changeover between phases of focusing (operating) and monitoring (checking performance standards, comparison, etc.). This implies that the spare–utilized capacity ratio is not constant but fluctuates across trials. Hence, as individuals engage in task operations, spare capacity is converted into utilised capacity, and the corresponding increase in task focus leads to a momentary decrease in monitoring. Failure to resume focusing would result in disturbances in the performance trajectory. In this way, a spare-utilised capacity threading view provides a natural framework for deliberating upon attention fluctuations in continuous tasks (Steinborn and Huestegge 2016, 2017, 2020).

A mere–effort account predicts a boost in mental focus and concentration (Steinborn et al. 2017; Wolf et al. 2025), as it encourages the instant mobilization of capacity by biasing the ratio between utilized and spare capacity in favour of the former (operating) over the latter (monitoring). Hence, socially-alerted cognition globally capacitates efficient information processing through increased focus on task-relevant (operating) relative to task-irrelevant processes (monitoring). Therefore, this account predicts a performance improvement globally. According to the event–distraction account (Baron et al. 1978; Sanders and Baron 1975), competition enhances readiness considered as the disposition to receive ongoing information from the social environment and the inclination to respond to it. In this way, competition makes the individual vulnerable to distraction because occasionally intensified monitoring makes attention likely to be captured by the onset of (social or non-social) events (Church 1962; Strauss 2002). However, this account does not specify the precise trigger conditions, frequency, or distribution of such events, nor how they are represented (Deutsch et al. 2023; Hasher and Zacks 1979; Hintzman 1988), making concrete predictions difficult. Distraction effects, when present, may appear in measures of performance variability and could be more pronounced under higher task difficulty.^1^

### Present study

The present study examined the impact of a two-person speeded arithmetic duel on both performance and subjective experience. A mixed within-subject design compared single-context and duel-context conditions (AA vs AB). This design enables a between-subject comparison of duel-context (..B) and single-context (..A) conditions, while the initial single-context (A..) phase serves to determine whether the two groups (drawn from the same population) are comparable or divergent. Arithmetic complexity (low vs high workload) was manipulated while retaining the speed-test nature of the task, thereby ensuring alignment with both automatic and controlled processing patterns. Typically, smaller problems (problem size < 10) are likely solved by automatic retrieval, whereas larger problems (problem size > 10) engage a mixture of retrieval and algorithmic operations (Ashcraft 1992; Blankenberger 2001; Gordon D. Logan 1988; Ratcliff 1978; Steinborn and Huestegge 2016, 2017, 2020). Our predictions were neutral, without favouring the mere–effort or event–distraction accounts. The mere–effort account predicts that the duel context enhances efficiency globally through increased motivation and focus. The event–distraction account predicts that competition can impair performance by increasing self-monitoring and attentional shifts towards the competitor, thereby elevating susceptibility to distraction. To assess experiential changes alongside performance, self-reported engagement, distress, and worry were measured before and after each session using the Dundee Stress State Questionnaire (DSSQ).

## Method

### Participants

A total of 114 volunteers (*M* = 21.8 years, *SD* = 3.5), primarily drawn from psychology courses, took part in the study. The sample contained more females than males, reflecting typical demographics in psychology programmes. All participants were in good health and reported normal cognitive and physical functioning. No specific recruitment criteria were applied beyond general availability and willingness to participate. An a priori power calculation, based on effect sizes reported for comparable effects in speed tasks (partial η^2^ ≈ 0.06–0.08), indicated that 34 participants per group would be sufficient to achieve 80% power at α = 0.05. The final sample comprised 50 participants per group, providing more than adequate statistical precision, particularly advantageous for the self-report measures.

### Ethical statement

Informed consent was obtained from all individual participants involved in the study. The research adhered to the 1964 Helsinki declaration and its subsequent amendments, or comparable ethical standards. The study was conducted in line with ethical guidelines for behavioural experiments and the national research committee of the German Research Foundation, and in line with the principles of the Ethikkommission des Instituts (Ethics Commission of the Institute) at Julius-Maximilians-Universität Würzburg, according to which the requirement for a formal ethical approval was waived as being deemed unnecessary.

### Task description

Task description. We used a paper‑and‑pencil mental addition verification task (Gordon D. Logan 1988; G. D. Logan 1990; Schumann et al. 2022a, b), to preserve the immediacy and social atmosphere of a face-to-face duel. Each item consisted of an addition problem with a proposed result, and participants worked through each sheet as quickly and accurately as possible. Correct results were verified by marking right, incorrect results were falsified by marking left. Easy problems had a problem size below 10 (e.g., 1 + 2 = 3, 4 + 5 = 9) and difficult problems had a problem size above 10 (e.g., 7 + 4 = 11, 8 + 9 = 17). Sheets of the same difficulty were presented in pure blocks, so that each bout comprised only easy or only difficult items.

### Task presentation

Each A4 sheet contained ten problems, and participants were instructed to work as quickly and accurately as possible. In the competitive setting, a simple “one error per sheet” rule (10%) was used as a pragmatic anchor, immediately understood without calculation, ensuring a common performance frame across participants. This threshold discouraged over-checking while avoiding strategic extremes, such as maximising speed at the expense of accuracy. In typical self-paced speed tests, error rates are already well below this level, so the allowance was deliberately generous. The fact that participants remained clearly under the limit further argues against deliberate sacrificing of accuracy for speed. The experiment comprised two sections (A: single-context; B: social-context), each containing four blocks (easy–hard–easy–hard), with each block consisting of five sheets. Completing five sheets constituted one round. An experimenter was present in all conditions. Completion time for each sheet served as the reaction-time measure; if needed, this could be divided by ten to yield an approximate per-item reaction time (Steinborn et al. 2018; Wühr and Ansorge 2020), although individual RTs were not strictly required for the planned analyses and interpretation.

### The math duel

The math duel. In the social-context condition, two participants competed in a head-to-head mental-addition duel. The match comprised four rounds, with one point awarded to the winner of each round and displayed on a scoreboard positioned in front of them. Thus, a match could end with scores such as 4:0, 3:1, or 2:2. Because the scoreboard was updated after every round, participants were continuously aware of their standing in the match. This setting shares core features with competitive dyads in sport research (Guldenpenning et al. 2017), where mutual visibility and real-time feedback generate a performance-relevant co-actor dynamic, even in the absence of deceptive intent.

### Self-report measures

We administered self-report measures before and after each of the experimental sessions. The Dundee Stress State Questionnaire (DSSQ), developed by Matthews et al. (2002), assesses the three fundamental dimensions of subjective state: engagement, distress, and worry. We used the short German version of the DSSQ (Langner et al. 2011, 2010), assessing the three facets on 5-point Likert-type rating scales.

### Design

We compared the social-context and single-context conditions in a mixed within-subject design with two groups: critical (AB) and control (AA). The critical group completed condition A (single context) followed by condition B (social context). The control group completed condition A twice. This arrangement allowed both within-subject and between-subject comparisons, enabling control over potential confounds and ancillary conditions (Heathcote et al. 2015, pp. 401–404).

### Experimental protocol

For the critical (AB) group, part A (single context) began with welcoming participants individually and assigning them to separate rooms, followed by the pre-task DSSQ, the single-context condition, and the post-task DSSQ. After a short break, part B (social context) was conducted in a room prepared for the math duel. Participants were seated side by side in front of a scoreboard, instructed about the duel format, and completed the pre-task DSSQ, the social-context condition, and the post-task DSSQ. The scoreboard was updated after each bout by a student assistant. The control (AA) group followed the same sequence, including the break and room change, except that both parts involved the single-context condition. The full session, including instructions, breaks, and questionnaires, lasted approximately 50 min.

## Results

### Data treatment

Data treatment. Mean reaction time (RTM) was computed as an index of average response speed, the reaction time coefficient of variation (RTCV) as a measure of relativised response-speed variability, and error percentage (EP) as the proportion of incorrect responses. To examine the distributional shape of responses, we calculated vincentized, interpolated cumulative distribution functions (CDFs) with ten percentiles for each experimental condition. In the present data, these distributions are based on sheet completion times (i.e., the time to complete a set of ten problems) rather than on individual item latencies. Percentiles were derived using an interpolation method, which estimates intermediate values between empirical ranks, allowing the construction of smooth percentile functions even when the number of observations per participant and condition is small. Because the AA and AB groups differ in session structure (alone–alone vs. alone–duel), the main effect of group conflates heterogeneous conditions. Accordingly, the inferential focus was placed on the Group × Session interaction rather than on the group main effect.

### Experimental effects on RTM

**Fig. 1:**
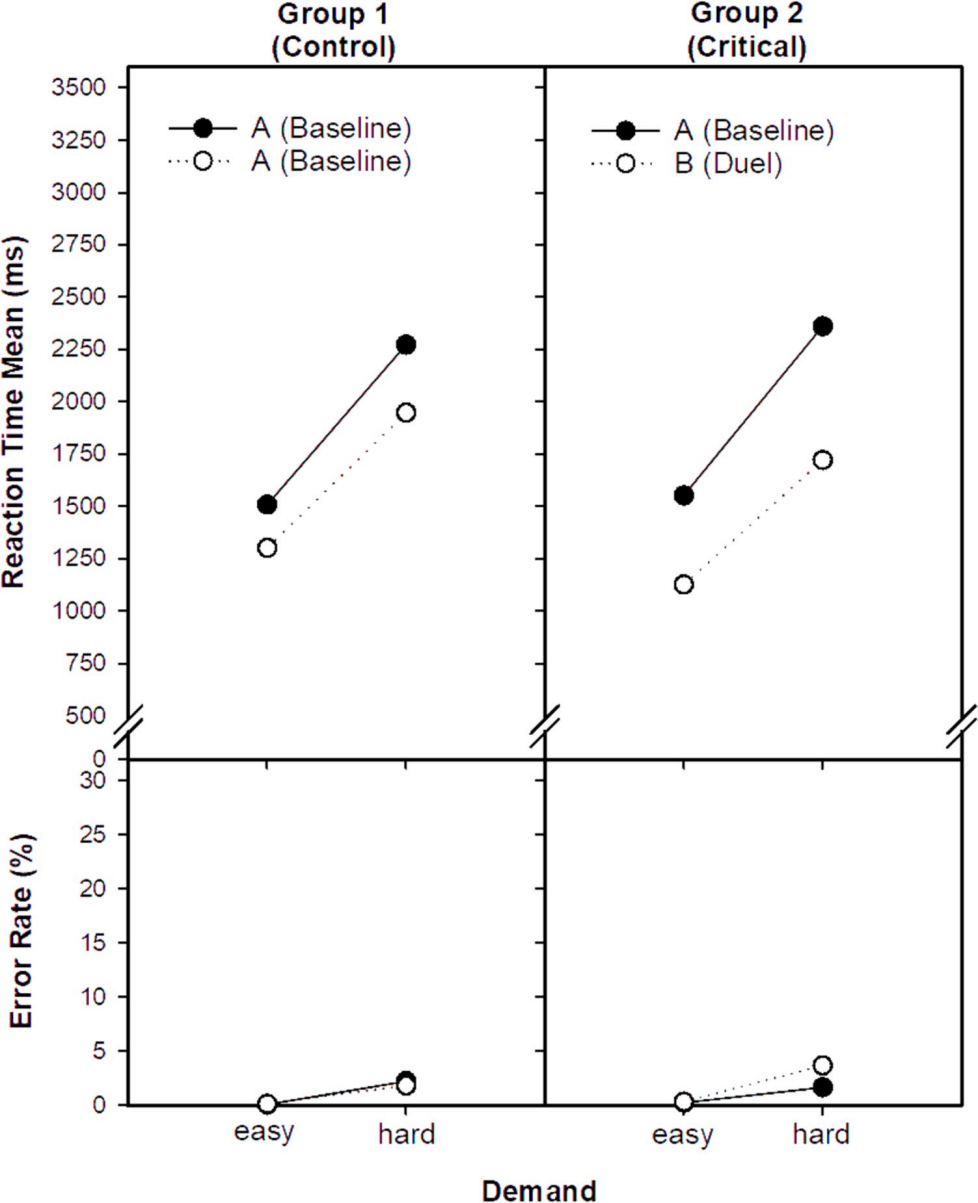
Reaction time mean (RTM) and error rate (ER) as a function of the factors group (controls vs. duel), session (session 1 vs. 2), and demand (easy vs. hard) in speeded mental arithmetic

**Fig. 2:**
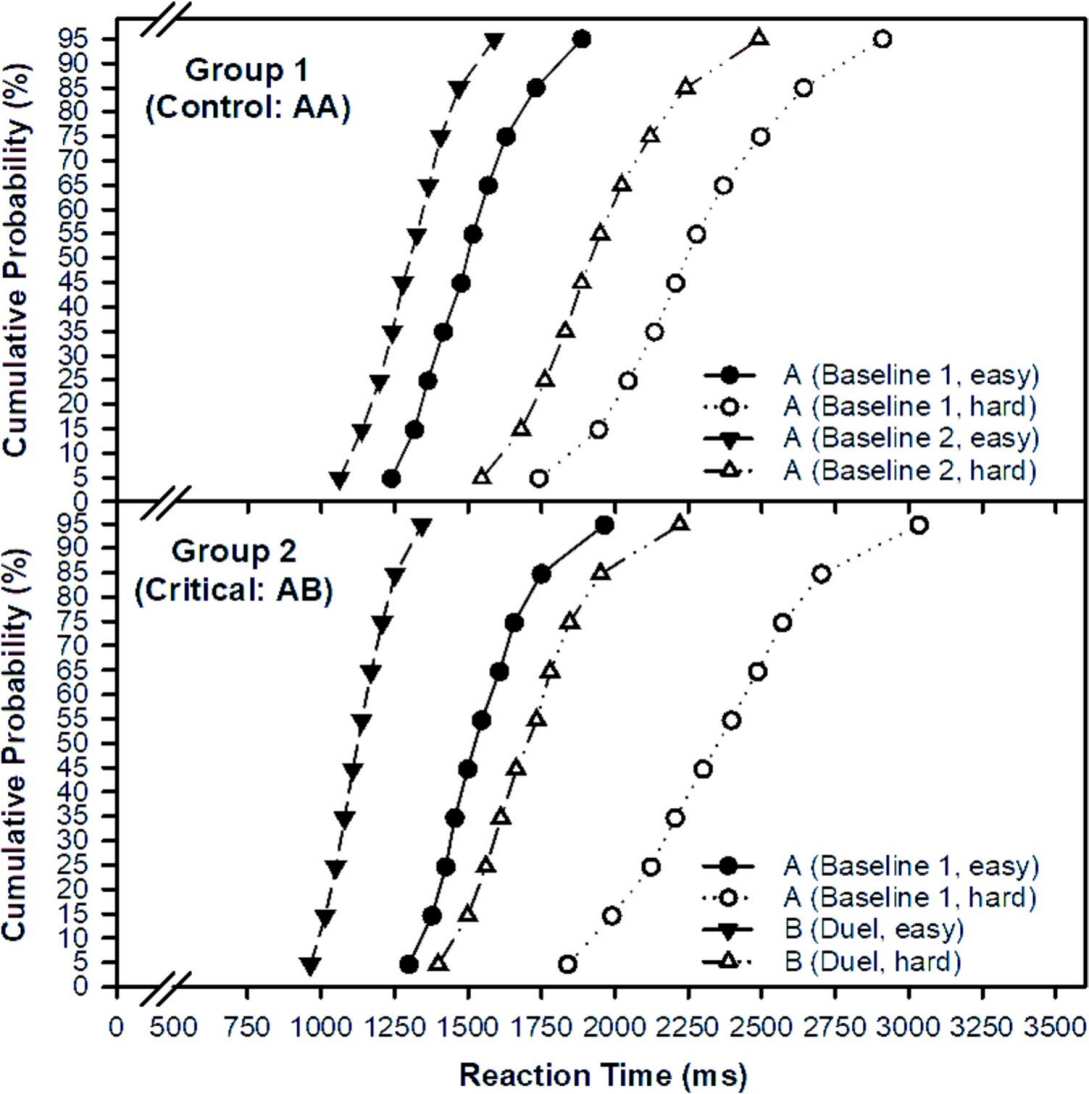
Vincentized interpolated cumulative distributive function (CDF) of reaction times for each combination of the factors group (controls vs. duel), session (session 1 vs. 2) and demand (easy vs. hard) in speeded mental arithmetic

RTM was analysed with a three‑factor mixed‑design GLM including group (AB vs. AA), session (first vs. second), and demand (easy vs. hard). Full statistics are given in Table 1 and visualised in Figs. 1, 2. The main effect of group is not meaningful in isolation because groups differ in session structure, therefore inference centres on the Group × Session interaction. There was a robust main effect of demand, with faster performance for easy than for hard problems, F(1,112) = 318.5, *p* < 0.001. Performance was also globally faster in the second session, F(1,112) = 419.3, *p* < 0.001. Critically, the Group × Session interaction was significant, F(1,112) = 46.8, *p* < 0.001, indicating a larger speed‑up in the critical group than in the control group. The Group × Session × Demand interaction showed a trend, F(1,112) = 2.8, *p* = 0.096, suggesting that the advantage for the critical group tended to be more pronounced for hard than for easy demand. In sum, competition accelerated responding for both difficulty levels, with a relatively stronger gain after the context change.

**Table 1:** Results of the mixed within-subject GLM: Experimental effects of the factors group, session, and demand on speeded mental arithmetic performance

Source			RTM			EP			RTCV		
		df	*F*	*p*	*η*^2^	*F*	*p*	*η*^2^	*F*	*p*	*η*^2^
*1*	Group	1,112	0.9	.352	.01	1.6	.206	.01	2.7	.103	.02
*2*	Session	1,112	419.3	.000	.79	6.7	.011	.06	9.2	.003	.08
*3*	Demand	1,112	318.5	.000	.74	53.0	.000	.32	23.1	.000	.17
*4*	Group × Session	1,112	46.8	.000	.30	13.5	.000	.11	1.7	.192	.015
*5*	Group × Demand	1,112	0.0	.965	.00	0.6	.436	.01	1.3	.258	.01
*6*	Session × Demand	1.112	33.2	.000	.23	5.1	.026	.04	0.3	.589	.00
*7*	Group × S × Demand	1,112	2.8	.096	.02	13.6	.000	.11	0.3	.572	.00

Effect size: partial *η*^2^*;* Experimental factors: Group (critical vs. control), Session (1 vs. 2), Demand (easy vs. hard mental arithmetic). RTM = reaction time (mean); ER = error rate; RTCV = reaction time coefficient of variation

### Experimental effects on EP

Complete statistics are reported in Table 1. The main effect of group is again not interpreted for the same design reason. Error rates were lower for easy than for hard problems, F(1,112) = 53.0, *p* < 0.001. Errors increased slightly from first to second session, F(1,112) = 6.7, *p* < 0.05. The Group × Session interaction was significant, F(1,112) = 13.5, *p* < 0.001, showing that this increase was confined to the critical group, whereas the control group showed little change. The Group × Session × Demand interaction was also significant, F(1,112) = 13.6, *p* < 0.001, indicating that the relative difference between groups was larger for hard than for easy demand. Although errors rose slightly under competition, rates remained low overall and clearly within the 10% allowance.

### Experimental effects on RTCV

Full results are in Table 1. Variability was lower for easy than for hard problems, F(1,112) = 23.1, *p* < 0.001, and decreased from first to second session, F(1,112) = 9.2, *p* = 0.003. There was no Group × Session interaction and no three‑way interaction, indicating that competition did not systematically alter relativised response‑speed variability beyond the global practice effect.

### Speed–error correlations

Speed–accuracy evaluation. The term speed–accuracy trade-off (SAT) refers to the potential inverse relationship between response speed and accuracy, where faster performance may come at the cost of reduced accuracy; however, this can occur at three distinct levels, each targeting a different aspect of performance:

*(a) Condition level: comparison of mean performance across experimental conditions.* This level addresses the classical SAT question: does the duel context produce faster responses at the cost of more errors? In our data, the duel context yielded faster responding and a small error increase, yet error rates remained far below the 10% threshold set by the instruction, indicating that overall performance stayed within acceptable limits.

*(b) Correlation level: covariation of speed and accuracy within a single condition*. This addresses whether faster individuals within the same context tend to be more error-prone (overall across all conditions: *r* = 0.18). In the single-context condition, RTM and EP were essentially uncorrelated (easy: *r* =  − 0.07; hard: *r* =  − 0.08), whereas in the duel context they correlated positively (easy: *r* = 0.43; hard: *r* = 0.25). This is the opposite of a classical SAT, indicating that in competition, slower individuals tended to commit more errors.

*(c) Effect level: relationship between individual change scores across contexts.* Here, each participant’s change in RT (ΔRT) from single to duel is plotted against the corresponding change in error rate (ΔEP; Fig. 4). This analysis revealed heterogeneity: most participants (≈ 89%) fell within a ± 2.5 percentage point error-change band, indicating negligible error change despite speed-up. A small minority (n = 4) showed the pattern consistent with a genuine trade-off (faster but sacrificing accuracy, Quadrant A), whereas some others slowed down and made more errors (Quadrant D, “choking under pressure”). The majority of duel participants improved speed without incurring notable accuracy costs, suggesting that the modest overall error increase reflects the probabilistic cost of reduced checking time rather than deliberate sacrificing of accuracy for speed.

### Introspective reports

**Fig. 3:**
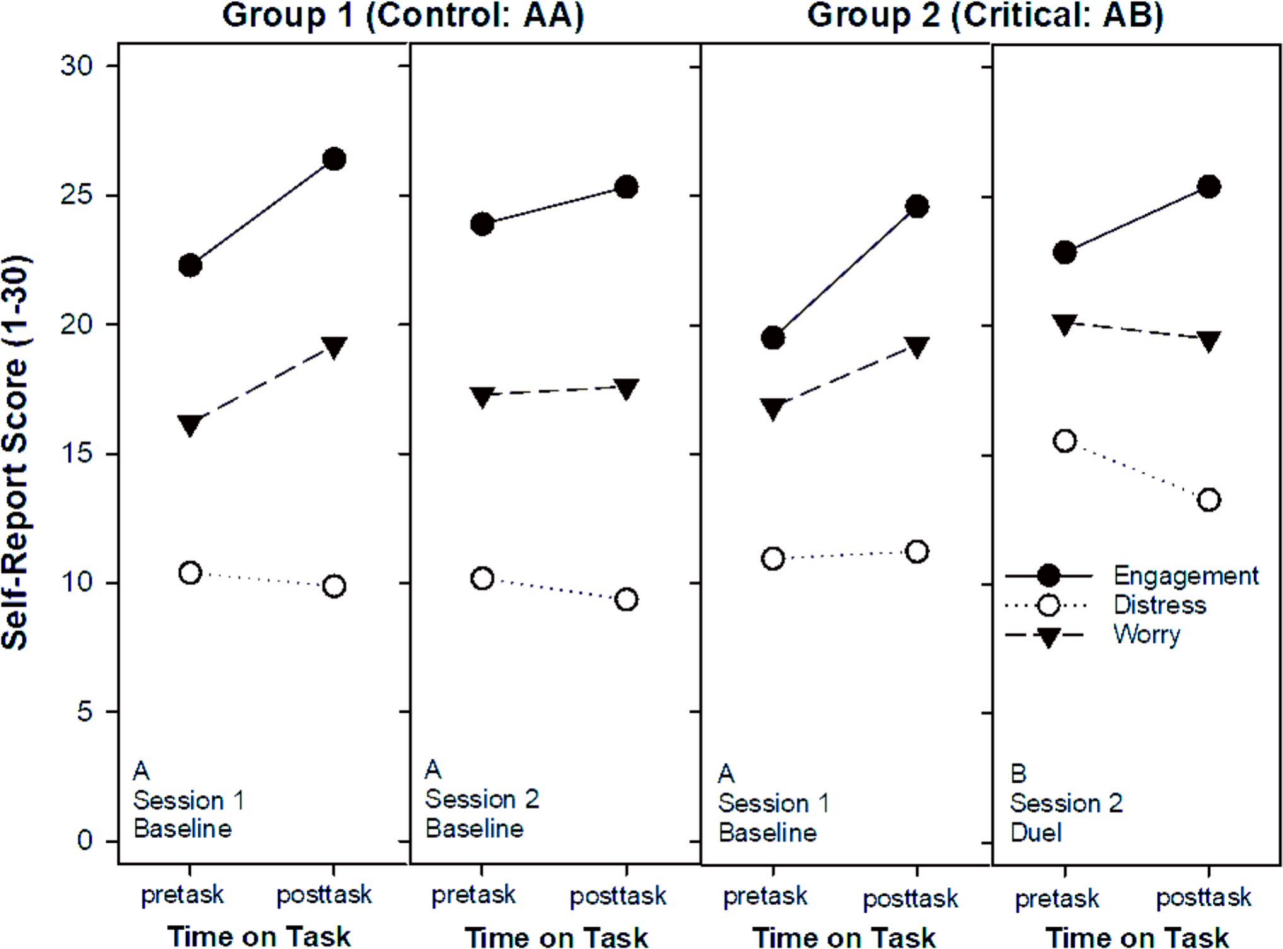
Fundamental dimensions of subjective stress state (task engagement, distress, and worry) as a function of the factors group (controls vs. duel), session (session 1 vs. 2), and time on task (pretest vs. posttest) in speeded mental-arithmetic performance

For each session, pre-task and post-task assessments of subjective state were collected, with primary focus on the task-engagement (motivation) score. Full statistical results are provided in Table 2 and illustrated in Fig. 3. The main effect of time-on-task (TOT) for engagement [F(1,109) = 115.5, *p* < 0.001] indicated that motivation increased, rather than declined, over the course of a session. The session × TOT interaction [F(1,109) = 16.1, *p* < 0.001] showed that this increase was larger in session 1 than in session 2. This pattern runs counter to the typical decline in motivation over time and is consistent with the stimulating nature of the task context, particularly in the initial encounter with the activity.

**Table 2:** Results of the mixed within-subject GLM: Experimental effects of the factors group, session, and time on task on self-report measures of the fundamental dimensions of subjective state

Source			Task engagement			Distress			Worry		
		df	*F*	*p*	*η*^2^	*F*	*p*	*η*^2^	*F*	*p*	*η*^2^
*1*	Group	1,109	3.3	.073	.03	15.3	.000	.12	11.0	.001	.09
*2*	Session	1,109	19.4	.000	.15	26.7	.000	.20	11.2	.001	.09
*3*	TOT	1,109	115.5	.000	.51	8.3	.005	.07	23.2	.000	.18
*4*	Group × Session	1,109	11.8	.001	.10	41.7	.000	.28	19.2	.000	.15
*5*	Group × TOT	1,109	2.9	.093	.03	0.3	.566	.00	2.2	.144	.02
*6*	Session × TOT	1.109	16.1	.000	.13	5.0	.028	.04	36.0	.000	.25
*7*	Group × S × TOT	1,109	0.0	.906	.00	3.1	.081	.03	0.0	.752	.00

Effect size: partial *η*^2^*;* Experimental factors: Group (critical vs. control), Session (1 vs. 2), Time on Task (TOT: pre-test vs. post-test)

## Discussion

### Summary

**Fig. 4:**
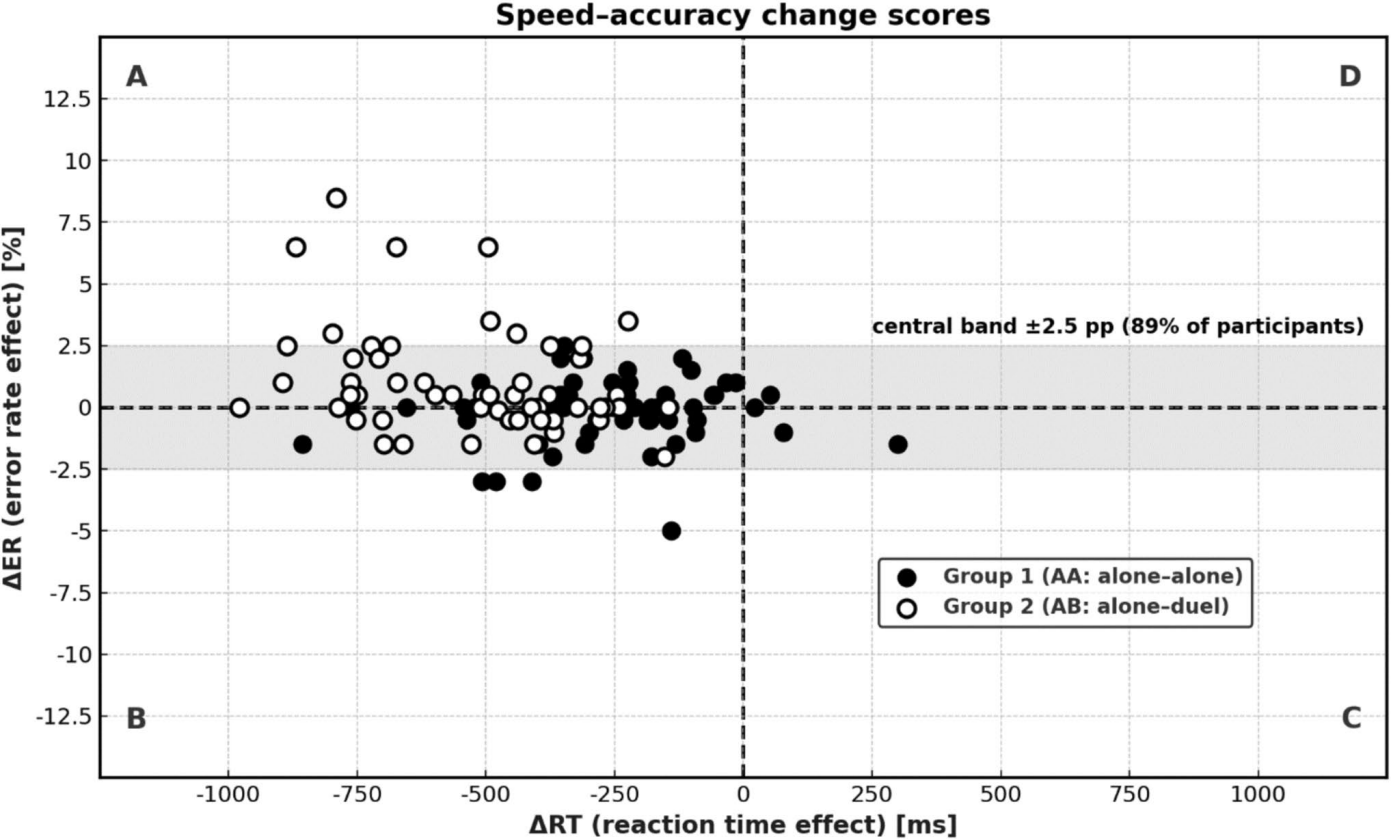
Individual change scores linking speed and accuracy. Each data point represents one participant. The x-axis shows the change in mean reaction time from Session 1 to Session 2 (negative values indicate faster responses), and the y-axis shows the corresponding change in error rate (positive values indicate more errors). Quadrants classify the joint direction of change: Panel **A**, trade-off (faster and more errors); Panel **B**, joint improvement (faster and fewer errors); Panel **C**, conservative shift (slower and fewer errors); Panel **D**, joint deterioration (slower and more errors). The shaded band indicates the central range (± 2.5 percentage points, 89% of participants)

The main findings can be outlined as follows: (1) Responses were faster in the duel-context (AB) than in the single-context (AA, control) condition, relative to baseline (Figs. 1, 2). This speed advantage applied to both easy and hard items, covering automatic and controlled processing; yet a closer inspection of the speed–accuracy trade-off requires differentiation across three analytical levels. (2) At the *level of experimental conditions*, participants in the duel-context responded faster but with somewhat higher error rates compared to the single-context control. This aggregate pattern suggests a modest shift along the speed–accuracy balance. (3) *At the correlational level,* we examined whether faster individuals were also more error-prone within a given condition. In the single-context condition, response speed and error rate were essentially unrelated. In contrast, in the duel-context condition, slower participants tended to commit more errors, which contradicts the standard assumption that faster responding is achieved at the cost of reduced accuracy. (4) *At the effect level* (Fig. 4), where individual change scores from baseline to duel were considered, performance adjustments proved heterogeneous. Most participants became faster without losing accuracy, some exhibited no clear change, and only a minority showed faster responses coupled with higher error rates. This heterogeneity indicates that the error increase observed at the condition level does not reflect a uniform strategic trade-off but a mixture of individual adjustment patterns. (5) Finally, contrary to the willpower-depletion view (Inzlicht et al. 2018), the duel-context did not reduce engagement or increase distress and worry relative to the single-context condition (Fig. 3). Instead, competition preserved, in some cases enhanced, the subjective experience of engagement (Belardi et al. 2022; Schmidt et al. 2024).

### Design methodology

Using an advanced design (Heathcote et al. 2015; Steinborn and Huestegge 2020), we examined the effects of a two-person duel in speeded arithmetic within a naturalistic context (Guerin 2009, pp. 12–29). The design incorporated both between-subject and within-subject contrasts of duel-context (AB) and single-context (AA) conditions, with the initial phase serving to establish baseline comparability. The results were that participants were faster but slightly more error-prone in the duel-context relative to the single-context condition. Our design thus offered several key advantages: First, the design establishes baseline comparability of the groups instead of merely assuming equivalence, making it possible to empirically verify and control potential sampling error. This provides a sounder basis for interpreting subsequent performance differences. Second, it contrasts duel performance with baseline within the same individuals, thereby reducing error variance compared to a purely between-group design and directly addressing residual sampling imbalances. Third, it controlled for mere-presence effects by keeping an experimenter present in both duel and single conditions, in this way, ensuring that any observed differences (in performance or self-report) could be attributed specifically to competitive interaction rather than to the simple presence of another person (Steinborn and Huestegge 2016, 2017, 2020).

The present design represents an improvement over common approaches in co-actor research, which typically rely on either purely between-subject or within-subject formats where conditions (A and B) are merely counterbalanced within one group. Our design addressed two critical limitations: sampling error in between-subject designs and order bias in within-subject designs. A counterbalanced one-group design (i.e., one half of participants: AB, the other: BA) has a critical shortcoming (cf. Heathcote et al. 2015, pp. 401–404; Steinborn and Huestegge 2020, pp. 1432–1433; Wajnerman-Paz and Rojas-Líbano 2022). If participants encounter competition first (BA), the subsequent control condition (A) may be contaminated by aftereffects of heightened engagement. The central problem is that the true effect cannot be disentangled from order-induced bias, which obscures their respective contributions. Though sequential counterbalancing is economical, it is unsuitable for studying social context because blockwise carryover effects compromise treatment purity (Sebanz et al. 2003; Vesper et al. 2011).

One final point concerns the replicability of our findings in comparable experimental settings. Successful replication does not depend on an exhaustive reproduction of microscopic procedural details, a practice that often generates narrow and convoluted debate, but rather on the identification and preservation of a few decisive factors that are logically evident and constitutive of the underlying mechanism (Zogmaister et al. 2024, p. 13). In our view, many reported replication failures do not reflect inconsistency of the phenomenon itself but a divergence in the conceptual framework from which studies are designed (Steinborn and Huestegge 2020; Wajnerman-Paz and Rojas-Líbano 2022, p. 7). In this way, we view replication not as the mechanical duplication of procedural steps but as the re-enactment of a theoretical construct within an empirical setting. If the construct is only loosely specified, ostensibly similar studies may in fact instantiate different conceptual objects, and then producing outcomes that are not genuinely comparable (Strack and Deutsch 2004; Stroebe and Strack 2014). It is therefore indispensable to specify clearly which design features are constitutive for the phenomenon under study and which are merely incidental. In our case, the construct defined by immediate, real-time interaction between participants. This must be distinguished from superficially related notions (e.g., rivalry, envy) which appear similar on the surface but arise from fundamentally different dynamics. By focusing on such constitutive features, replication efforts can target the same conceptual object, ensuring that divergent findings become scientifically informative rather than reflecting only technical differences in procedure.

While our mixed within-subject structure safeguarded baseline referencing and minimised order bias, the critical innovation was the creation of an authentic co-actor environment. Many previous studies relied on simulated competitors presented on a screen, which tend to elicit weak involvement and ambiguous commitment. In contrast, our face-to-face arithmetic duel, with direct visibility and real-time scoring, generated a concrete social encounter that sustained coordinated engagement (Klaffehn et al. 2018; Pedraza-Ramirez et al. 2020). The ecological validity here did not stem from mimicking classroom practice, but from instantiating a compelling sense of social presence. Participants experienced themselves as active agents in a live duel, which heightened immersion and sharpened competitive motivation within laboratory constraints. In this way, the duel setting provided a psychologically robust and ecologically grounded realisation of co-actor dynamics, enabling us to capture both performance and motivational consequences of real-time interpersonal engagement.

*Fourth,* it is essential to employ tasks with high measurement precision, since reliability in speeded performance depends on item characteristics, trial numbers, and trial sequencing (Miller and Ulrich 2021, 2022). Several studies have neglected these aspects by using arbitrary tasks or too few trials, reducing measurement accuracy, whereas our task adhered to these principles and thus ensured stable and interpretable outcome measures. *Fifth*, we introduced a clear contrast between easy and difficult arithmetic tasks, while keeping both within the framework of speed tests. This manipulation is theoretically motivated: easy problems (sums < 10) are typically resolved through direct retrieval, whereas more difficult problems (sums > 10) require algorithmic operations or mixed retrieval–computation strategies (Blankenberger 2001; Guldenpenning et al. 2017; Vandierendonck et al. 2010). This contrast allows a dissociation between automatic and controlled processing, forming the basis for interpreting how competitive engagement modulates performance across varying levels of cognitive demand (Anderson 2021; Oberauer 2019, 2024; Steinborn and Huestegge 2016, 2017, 2020; Wolf et al. 2025).

### Theoretical implications

The event-distraction model (Baron et al. 1978; Sanders and Baron 1975) predicts that competition elevates arousal, increases susceptibility to irrelevant input, and thereby causes intermittent disruptions in controlled processing. At first glance, the duel effect of faster responses with slightly more errors might seem to support this account. We consider this conclusion unconvincing. Errors increased only marginally and remained well below the 10% allowance set by the duel rules (hence not diagnostic of criterion relaxation). Relativised response-speed variability (RTCV) did not rise beyond practice, indicating stable control rather than increased fluctuation. *At the correlational level*, it were the slower individuals that were the more error-prone in competition, the opposite of a speed–accuracy trade-off. Finally, individual change scores (ΔRT, ΔER) revealed marked heterogeneity: some participants became faster without losing accuracy, others showed little change, and only a minority displayed genuine speed–accuracy deterioration (Fig. 4). Thus, the error increase observed at the condition level (Fig. 1) does not signal a uniform strategic trade-off but reflects a mixture of distinct adjustment patterns. In this light, the data do not support the idea of a general distraction-driven impairment. For most participants, responses became faster while accuracy remained essentially stable, with minor deviations amounting only to incidental slips. Only a small minority showed escalating errors alongside speed gains, a pattern more consistent with a genuine speed–accuracy trade-off, yet one that did not *typify* the group as a whole.^2^

The mere-effort model (Harkins 2006; Steinborn and Huestegge 2020) aligns more closely with the overall result pattern. It states that competition, insofar as it provides a clear goal structure, enhances motivation and shields task focus against distraction and mind-wandering. Its prediction (faster responding with few errors) is best understood as a symbolic idealisation: theories abstract from empirical impurities and describe an archetypal relation rather than a literal performance profile. When applied to chronometric tests, however, this idealisation requires adjustment, because faster responses necessarily shorten the available processing time and can increase the chance of occasional errors. A response speed-up necessarily reduces target processing time, which as a direct mathematical consequence can slightly increase the likelihood of occasional errors, even without the explicit intention to relax accuracy criteria. Formal decision models capture this logic (Brown and Heathcote 2008; Cao et al. 2020; Steinborn et al. 2017): when response criteria are lowered, either by explicit instruction to try harder or by advance knowledge of task ease, responses are pre-allocated to a condensed processing time. This entails both stronger focus and greater urgency, but at the cost of reduced time for information accumulation. Even with increased effort and improved efficiency, compressed processing time can still produce some minor error costs without directly implying a strategic trade-off.

Theoretical models gain their explanatory power not from matching individual data points one by one, but from their ability to generalise across contexts. The mere-effort account illustrates this: its claim that competition yields faster responses without accuracy loss is not meant as a dataset-specific rule, but as a formal statement about motivational regulation. The problem is to translate such symbolic formulations into measurable indicators, a step that can bias interpretation depending on the chosen measure. In experimental psychology, the conventional starting point is group means (Fig. 1). Means are convenient entry points though often mistaken for direct evidence of an underlying mechanism. In fact, they provide only a preliminary hint that further analysis is needed. We argue that real explanatory progress comes from examining individual performance patterns and grouping them into recurring adjustment types. In our case, three types emerged: most participants became faster without losing accuracy, some showed diffuse change without clear direction, and only a small minority displayed a clear speed–accuracy sacrificing pattern (Fig. 4). Thus, the error increase at the mean level does not reflect a uniform mechanism but represents the blended outcome of heterogeneous individual strategies (Brown and Heathcote 2008; Bruning et al. 2022; Estes 1956; Gutzeit and Huestegge 2025; Haaf and Rouder 2019).^3^

## Final conclusion

Although the present findings align more closely with a mere-effort than with an event-distraction account, no single experiment can adjudicate between theoretical propositions that abstract beyond specific task formats and measures. The contribution of this study is not to close debate with final answers but to open pathways for further inquiry. Two such routes emerge from our duel-cognition paradigm. First, analyses of speed–accuracy relations must move beyond aggregate means. This is not only specific to the present case but represents a more general requirement: progress depends on shifting from group averages to individual strategy profiles. Participants may adopt different strategies, some maintain a stable approach, others follow a consistent alternative, and still others alternate between strategies across the task. When such heterogeneous patterns are collapsed into a single mean, the result is an artefactual mixture rather than a genuine mechanism. Future work must therefore identify and cluster recurring strategy types of effort mobilisation, and only then derive averages that reflect systematic behaviour. Second, the paradigm raises the issue of ecological validity. A duel-like competition may engage dynamics that screen-based or simulated opponents cannot capture. A central task for future research will be to determine when genuine human–human interaction is indispensable and when computer-simulated or artificial agents can serve as valid proxies (Robison and Nguyen 2023). The present study should therefore be read not as an endpoint but as a blueprint: it shows how live competition can be integrated into chronometric designs to refine theories of motivational engagement under socially alerted cognition (Guldenpenning et al. 2017; Pedraza-Ramirez et al. 2020; Steinborn and Huestegge 2020).

## Supplementary Information

Below is the link to the electronic supplementary material.

Supplementary file1 (CSV 16 KB)

Supplementary file2 (SAV 16 KB)

Supplementary file3 (TXT 5 KB)

## Data Availability

The data that support the findings of this study are included within the article and its supplementary files.

## Footnotes

1 One reviewer asked whether the event-distraction account can be dissociated from a mere-effort account, and whether the assumptions about meta-cognitive monitoring should be tested directly. While such a definite dissociation is indeed difficult (and may in practice be impossible), a probative test would require a systematic experimental manipulation of the event stream (distributions, local sequences, a priori and conditional probabilities of social cue events, task cue events, etc.) together with outcome measures that are sensitive to anchoring by recent context. Deutsch et al. (2023) applied precisely this type of methodological logic though situated in a different research domain, in which participants were presented with controlled and experimentally varied event sequences and asked to provide comparative judgments. Relevant to the present context is the demonstration that systematic manipulations of distributions and sequences reliably expose anchoring and order effects. This illustrates the kind of approach that would, in principle, provide the methodological leverage to disentangle the event-distraction account from a mere-effort account. Translating such a framework to co-actor designs would indeed be highly informative (and ultimately the most conclusive way to test the distinction), yet it would require multiple groups, factorial control of event distributions, and measures of both performance and representation. In our task the events of interest are task-irrelevant (hence such a programme would constitute a separate line of research rather than an add-on to the present study). Moreover, competitive settings introduce additional complexity by modulating arousal (which can change event salience and memorability), implying that concurrent state measures would need to be collected before and after testing.

2 One reviewer argued that the performance benefit observed in a dueling context may not indicate increased effort but rather a more elementary decision to respond faster, for example by lowering response criteria. We interpret this not as an alternative interpretation but as one specification of the effort construct itself, as a subordinate expression within its broader regulatory domain. In our view, effort in speeded tasks is represented by three operative components: increased focus (Anderson 2021; Janczyk & Kunde 2010, 2020; Oberauer 2019, 2024; Theeuwes 2025); maintenance of engagement (Steinborn et al. 2017; Strayer et al. 2024; Wolf et al. 2025); and noetic coordination, that is, the orchestration of timing and processing overlap in balanced form. This last component is particularly relevant in complex arithmetic or dual-response contexts, where performance improvement requires reorganisation of cognitive operations, since excessive reliance on response urgency alone may destabilise performance. A pure model of urgency would not sustain performance, since it neglects both the stabilising role of focus and the regulatory role of coordination. Thus, the reviewer’s suggestion can be understood as a specific expression within the larger construct of effort, not as a replacement of it.

3 One reviewer questioned whether our results should be read as a conventional speed–accuracy trade-off. This view is understandable: faster responding combined with a rise in errors is often interpreted this way (indexed through group-level means). Yet this interpretation presupposes that means adequately represent the underlying process, which is only the case if all participants adopt essentially the same behavioural strategy. If so, individual variation would reflect minor fluctuations within a common pattern, making the mean representative. In our data, however, change scores revealed three distinct adjustment types: most participants became faster without losing accuracy, a second subset showed little systematic change, and a small minority displayed speed gains accompanied by marked error increases. Under such circumstances, the mean merges qualitatively distinct strategies into an artefactual composite. So what appears as a uniform trade-off at the condition level might be better understood as a heterogeneous mixture (Fig. 4). The likelihood of errors may rise by mathematical necessity when processing time is reduced, since less information can be accumulated, and we thus interpret the duel-benefit not as intentional sacrifice of accuracy but as evidence for intensified coordination (Brown & Heathcote 2008, pp. 158–160; Heitz 2014, pp. 9–11; Ruthruff 1996, pp. 56–64; Steinborn et al. 2017, pp. 140–142).
